# The Power of First Impressions: Can Influenza Imprinting during Infancy Inform Vaccine Design?

**DOI:** 10.3390/vaccines8030546

**Published:** 2020-09-19

**Authors:** Melissa Rioux, Mara McNeil, Magen E. Francis, Nicholas Dawe, Mary Foley, Joanne M. Langley, Alyson A. Kelvin

**Affiliations:** 1 Department of Microbiology and Immunology, Faculty of Medicine, Dalhousie University, Halifax, NS B3H 4R2, Canada; melissa.rioux@dal.ca (M.R.); mara.mcneil@dal.ca (M.M.); m.francis@dal.ca (M.E.F.); nc350339@dal.ca (N.D.); mr471027@dal.ca (M.F.); 2 Vaccine and Infectious Disease Organization-International Vaccine Centre (VIDO-InterVac), Saskatoon, SK S7N 5E3, Canada; 3 Department of Pediatrics, Division of Infectious Disease, Faculty of Medicine, Dalhousie University, Halifax, NS B3K 6R8, Canada; joanne.langley@dal.ca; 4 The Canadian Center for Vaccinology (IWK Health Centre, Dalhousie University and the Nova Scotia Health Authority), Halifax, NS B3K 6R8, Canada; 5 Department of Community Health and Epidemiology, Faculty of Medicine, Dalhousie University, Halifax, NS B3K 6R8, Canada

**Keywords:** influenza virus, immune response, infant immunity, imprinting, *Orthomyxoviridae*, vaccination, influenza vaccines

## Abstract

Influenza virus infection causes severe respiratory illness in people worldwide, disproportionately affecting infants. The immature respiratory tract coupled with the developing immune system, and lack of previous exposure to the virus is thought to synergistically play a role in the increased disease severity in younger age groups. No influenza vaccines are available for those under six months, although maternal influenza immunization is recommended. In children aged six months to two years, vaccine immunogenicity is dampened compared to older children and adults. Unlike older children and adults, the infant immune system has fewer antigen-presenting cells and soluble immune factors. Paradoxically, we know that a person’s first infection with the influenza virus during infancy or childhood leads to the establishment of life-long immunity toward that particular virus strain. This is called *influenza imprinting*. We contend that by understanding the influenza imprinting event in the context of the infant immune system, we will be able to design more effective influenza vaccines for both infants and adults. Working through the lens of imprinting, using infant influenza animal models such as mice and ferrets which have proven useful for infant immunity studies, we will gain a better understanding of imprinting and its implications regarding vaccine design. This review examines literature regarding infant immune and respiratory development, current vaccine strategies, and highlights the importance of research into the imprinting event in infant animal models to develop more effective and protective vaccines for all including young children.

## 1. Introduction

**Figure 1:**
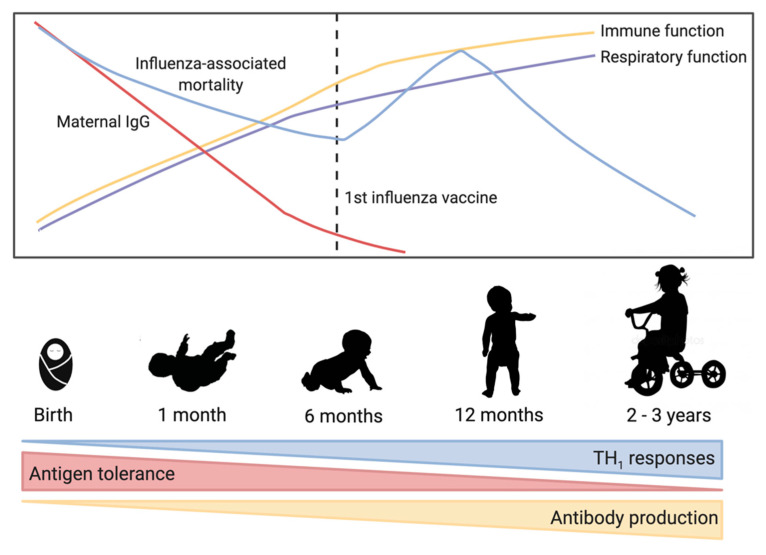
Trends in early-life immune development and influenza virus-associated mortality. As immune and respiratory function improve over the first few years, influenza-associated mortality decreases. Maternal immunoglobulin G (IgG) is high in full term babies but then decreases rapidly over the first few months of life. At birth, the immune system is biased toward T helper cell type 2 responses, but maturation over the first 2–3 years of life is characterized by increased T helper cell type 1 responses and increased antibody production allowing a balanced response to antigens.

Influenza virus is a negative-sense RNA virus and a major burden on global health [1]. Influenza viruses mutate rapidly due to antigenic drift and new strains emerge each year leading to continual disease in humans [2]. Periodic antigenic shift can lead to major changes in the influenza virus and lead to pandemics. Influenza virus types A and B currently circulate in humans, and are estimated to infect between 5–10% of adults and 20–30% of children each year [3]. Clinical symptoms of influenza virus infection can vary from mild upper respiratory tract symptoms to complicated pneumonia and possibly multi-organ failure and death [4]. Influenza virus complications are most frequent in children and the elderly [4,5], with some of the highest hospitalization and death rates due to influenza occurring in these ages groups [6,7]. Figure 1 highlights some of the important trends in early immunology and influenza virus-associated mortality.

There infant immune system differs from older children and adults. These differences include variant levels of soluble immune factors [8,9], lower levels of antigen presenting cells [10], increased Th2 responses [11], and lower levels of immunoglobulins [12,13]. Moreover, immunization of pregnant mothers has also been shown to influence the immune responses of their unborn children [14]. Although not covered here, investigation of how maternal immunization may influence imprinting is an important research priority. These unique immune system characteristics may also contribute to the different vaccination outcomes after split-virion influenza vaccine immunization experienced in infants and young children compared with adults. Furthermore, infants are more likely to be naive to influenza viruses which alters disease susceptibility and vaccine responses.

Influenza viruses mutate rapidly over time leading to new seasonal virus strains that can evade pre-existing immunity, the antigenics in the annual influenza vaccine are updated annually. There are several vaccine platforms currently available and approved for use including inactivated (IIV), live attenuated (LAIV), recombinant, and adjuvanted vaccines [15]. Only IIV are authorized for children aged from six months to two years, and no vaccine is authorized for use earlier than six months of age [15]. Figure 1 highlights some of the important trends in early immunology and influenza virus-associated mortality.

One’s first exposure to influenza virus, whether that be by infection or vaccination, is a significant event which we refer to as the *imprinting event* [16]. In particular, the first influenza virus infection shapes the immune system with respect to influenza virus antigens, and thus defines the immune response in subsequent influenza virus infections and vaccinations [16]. More work is needed to understand the full extent to which vaccination of infants imprints the immune system compared to infection. Understanding the imprinting event in the context of the infant immune system will be important in designing more effective vaccines to protect this vulnerable population.

Researchers have used several infant animal models to study the infant immune response to influenza virus infection, including mice and ferrets. A literature review of infant mice studies has shown decreased T cell immune responses in infant animals [17,18], but studies investigating humoral and antibody responses are currently lacking. Reviewing literature from infant ferret studies has demonstrated that outcomes of influenza virus infection may be significantly dependent on ferret age post-partum [19,20,21,22]. These models, discussed in detail below, may be useful for future studies involving imprinting and development of immune memory so that infant-specific vaccines can be developed.

Below, we review the literature surrounding infant immune imprinting in the context of influenza vaccination. We will first discuss influenza virology, clinical outcomes, and vaccine platforms. We will then discuss how this pertains to infant infection and vaccination. Finally, we will end with a discussion of possible animal models for further investigation of infant influenza virus immunity and vaccination.

## 2. Influenza: The Virus, Disease, Vaccine and Children

**Figure 2:**
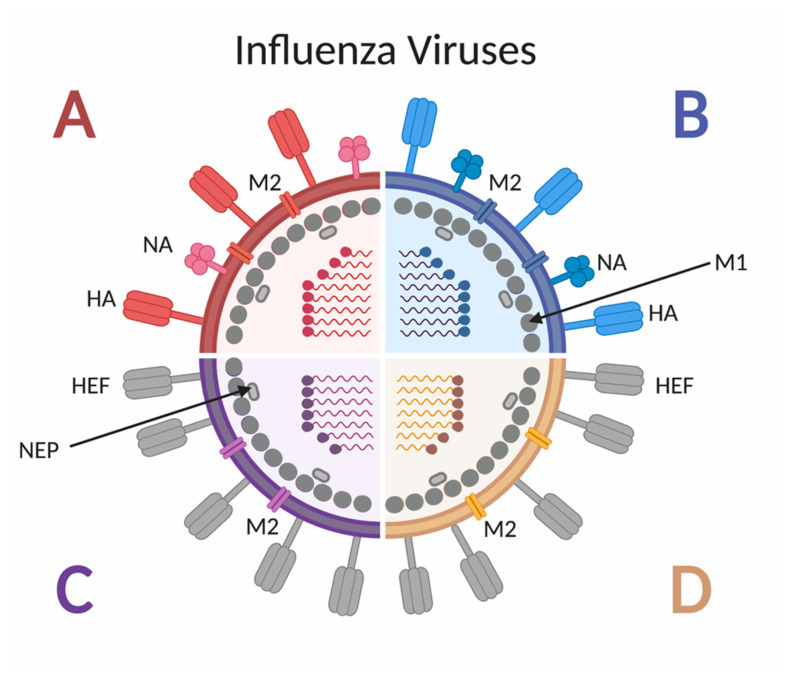
Schematic of influenza A, B, C and D virus structure. Influenza A and B viruses express surface glycoproteins hemagglutinin (HA) and neuraminidase (NA), as well as the M2 ion channel. Both A and B viruses have 8 genomic segments coding for at least 10 proteins. Influenza C and D viruses express the surface glycoprotein hemagglutinin-esterase fusion (HEF), as well as the M2 ion channel. Both C and D viruses have 7 genomic segments coding for 9 proteins. All four types of influenza viruses express the M1 protein along the inner surface on the envelope, adjacent to the nuclear export protein (NEP).

Influenza viruses are negative-sense RNA viruses belonging to the *Orthomyxoviridae* family [23]. As shown in Figure 2, there are four types of influenza viruses, but only influenza A virus (IAV) and influenza B virus (IBV) cause seasonal epidemics in humans [24], and only IAV has historically caused pandemics. Influenza A subtypes are classified by the glycoproteins hemagglutinin (HA) and neuraminidase (NA) found on the outer viral membrane [25]. To date, 18 different HA subtypes and 11 different NA subtypes have been identified [25], with the H1N1 and H3N2 subtypes currently circulating in humans, although other subtypes have been shown to cross species barriers and infect people [26]. The HA proteins can be grouped by amino acid similarity in the membrane proximal domain of the protein: group 1 includes H1, H2, H5, H6, H8, H9, H11, H12, H13, H16, H17 and H18 and group 2 includes H3, H4, H7, H10, H14, H15 [27]. Due to the various combinations of subtype and strain exposures and the continual shift and drift of the viruses [28], unique immune histories are created per individual.

The pathology caused by seasonal influenza virus infection is primarily localized to the respiratory tract, where the virus infects respiratory epithelial cells. Damage to the respiratory tract is mainly caused by the host inflammatory response to the virus Symptoms of infection can range from mild upper respiratory symptoms to lower respiratory tract involvement manifested by bronchitis, bronchiolitis, and/or complicated pneumonia [29]. Clinical presentation can vary depending on age, underlying co-morbidities and to some extent severity may vary according to the virus strain. Some influenza viral strains are able to infect both the upper and lower respiratory tract, while other viruses remain only in the upper [29]. The tropism of the virus often dictates disease, as viral infection in the lower respiratory tract can lead to compromised lung function. Some influenza viral strains are able to infect both the upper and lower respiratory tract, while other viruses remain only in the upper [30]. Approximately 30–40% of hospitalized patients with laboratory-diagnosed influenza also develop acute pneumonia, which is overrepresented by patients below 5 years and above 65 years of age [31]. Diffuse alveolar damage is possible, which can lead to respiratory dysfunction, endothelial leakage, precipitating in multi-organ failure and even death [32]. Respiratory failure may occur due to airway obstruction, loss of alveolar structure, degradation of the lung extracellular matrix, and epithelial cell death [29].

Although annual influenza vaccination is not implemented in the majority of countries across the world, it is recommended in many high income countries as the most important means to prevent infection by emerging seasonal influenza strains [33] and has been promoted by the Centers for Disease Control and Prevention since 2010 [34]. Importantly, vaccine immunogenicity and efficacy are highly dependent on the host’s age. The elderly and infants both have decreased responses to vaccination. The purpose of an influenza vaccine is to deliver viral antigens, typically the HA protein from the predominant strains of the season. Historically, the influenza virus was chemically inactivated following harvest from embryonated chicken eggs to produce whole-virion inactivated vaccines [35]. However, split-virion or subunit vaccines are now more commonly used since they cause less reactogenicity than whole virus vaccines after administration [36]. There are several influenza vaccine platforms currently in use including inactivated influenza vaccine (IIV), live-attenuated influenza vaccine (LAIV), recombinant, and adjuvanted influenza vaccines. The recommended influenza vaccine platform differs by age group, as shown (Table 1). Specific to infants and young children, the inactivated trivalent (TIV) and quadrivalent vaccines (QIV) are available for those aged from 6 months to 2 years and LAIV for those from 2 to 17 years. It is hypothesized that the direct immunization with LAIV in the respiratory mucosa is the mechanism driving immune responses in the younger age groups. Since infants and children have minimal pre-existing or no immunity due to low influenza virus infections or exposures, the LAIV may serve as a mechanism for imprinting the respiratory mucosa. An adjuvanted vaccine may also serve as a mechanism for inducing great immune responses in children. Three of the most common adjuvants used in influenza vaccine formulation studies include alum or aluminum salts and the two oil-in-water emulsions MF59 and AS03 [37]. Although there are several oil in water adjuvants in development, only MF59, is authorized for use in Canada and has been specifically shown to increase responses in children [38]. Moreover, FLUAD^TM^ pediatric which has MF59 as an adjuvant is approved for use in children [39].

## 3. The Predictive Power of Influenza Virus Immune Imprinting

In order to elicit the best immune response at vaccination, we need to consider how the influenza virus first imprints the immune system, how memory is established, and subsequently how the imprinted immune system is recalled over a lifetime. Evidence suggests that due to the continued presence of influenza virus strain specific antibodies, memory B cells produced during early exposures to the influenza virus still circulate in adult life [41]. The concept that one’s first influenza virus exposure in early life affects subsequent immune responses to infection and vaccination has been described for decades. The term original antigenic sin (OAS) was coined in the 1950s by Thomas Francis to describe the dominating effect of pre-existing immunity on the immune responses and the outcomes of infection later in life [42]. The “sin” of OAS refers to the preference of the immune system to recall pre-existing antibodies stimulation by cross-reactive epitopes on memory B cells instead of eliciting new ones against a novel antigen during the current infection [12]. Thereby, the older memory B cells are stimulated to elicit antibody production where the antibodies are specific to older influenza viruses and not to specific epitopes on the new viruses. The negative connotation of “sin” in OAS highlights the possible deleterious effect of the first infection on subsequent immune responses as pre-existing immunity may eclipse the needed responses during the current infection. As more studies are being done investigating the effects of pre-existing immunity, the phenomenon of pre-existing immunity influence has also come to be referred to as antigenic seniority, negative interference, back-boost, and negative antigenic interaction [43,44,45,46].

Our group refers to the first exposure to influenza virus and the resulting development of initial influenza immune memory as the *imprinting event* [28] which was first described as such by Gostic and colleagues [47]. Since evidence suggests that imprinting can be both beneficial or deleterious to the outcome of subsequent influenza infections and vaccination events, we feel imprinting more accurately describes the effects of the first influenza exposure as opposed to the negative effects implied by terms such as OAS. The imprinting phenomenon has been demonstrated repeatedly in studies where age cohorts are differentially impacted by emerging influenza viruses. For example, elders were least affected by the 2009 H1N1 pandemic in terms of developing severe disease leading to mortality. These effects were hypothesized to be due to childhood imprinting following infection with an antigenically similar virus from the 1918 pandemic. Immune memory was able to be recalled during exposure to the 2009 H1N1 virus leading to protection [48,49]. Moreover, a report by Worobey and colleagues gave evidence that pre-existing immunity from circulating H1N1 strains may have provided some protection from the 1918 pandemic strain which was estimated to cause between 50 and 100 million deaths worldwide [50]. However, in the same article, the authors suggested that individuals imprinted by an H3N2 strain fared worse. From data such as this we are able to cross-reference a person’s birth year with the strain that was circulating to almost predict how they may respond to a contemporary strain of influenza virus based on antigenicity between the strains [47].

In the seminal imprinting study by Gostic and colleagues in 2016, the HA stalk imprinting hypothesis was put forth. This hypothesis specifically contended that cross-protection could occur if individuals were imprinted with an influenza virus containing an HA protein belonging to the same HA group as a subsequent infecting strain [47]. For example, H1 and H5 influenza proteins belong to the group 1 HA designation while H3 and H7 belong to group 2. If an individual was imprinted by an H1 virus in childhood, the hypothesis suggests that they would then be protected from severe disease if exposed and infected with the highly pathogenic H5N1 virus. The mechanism of this protection is thought to be the ability of antibodies directed toward the stem of the H1 molecule to cross-react with the stem of H5 molecules blocking infection and leading to protection from severe H5N1 disease. Since conserved regions exist on the HA stem of each HA group, cross-reactive antibodies would be produced by restimulated older memory B cells that were initially established during the primary infection. Additionally, this study also showed that those who were imprinted with H3 strains which would have been circulating during their birth year, would not be afforded cross-protective responses against an H5 virus. More recently, Arevalo et al. showed that natural H1N1 imprinting in childhood was able to elicit HA stem specific antibodies that were recalled at reinfection and unable to bind efficient to the reinfecting strain suggesting an imprinting event specifically on HA stem epitopes [51]. Although a report by Tesini and colleagues indicated that the antibody and memory B cell responses during vaccination in a previously infected host is dynamic with several outcomes [52]. Specifically, this work has shown that correlations with the expansion of memory B cells and elicited antibodies were reactive toward the antigenically variable head and conserved stem. Moreover, the antibodies reactive to the variable HA head were specific to both the newly infecting virus as well as other viruses which the individual came into contact with in the past and may cause antigenic sin type responses. Imprinting has also been shown to impact vaccine effectiveness. A study by Skowronksi et al. showed an age-specific decrease in vaccine effectiveness in a non-elderly cohort which they hypothesized to be due to the imprinting in that cohort with a mismatching H3N2 clade virus to that in the vaccine [53]. Upon vaccination against H3N2 viruses in 3C. 2a, an antigenically distinct clade than the circulating clade from their birth year, the authors proposed based on their ‘I-REV’ (imprint-regulated effect of vaccine) hypothesis that exposure to an antigenically distant virus interfered with the imprinted immune response which would have protected this cohort [16,53]. Moreover, our group has also shown that vaccine outcome can be significantly influenced experimentally. In a preclinical study, imprinting ferrets with a historical H1N1 influenza virus which was antigenically distinct from vaccine components and a challenge virus led to differing vaccine responses in comparison with naive vaccinated ferrets [54]. Our data suggested that memory B cells were able to be recalled at the time of vaccination.

Taken together, these descriptive studies show that influenza outcome as well as vaccine effectiveness may be inferred by analyzing what influenza strain was circulating during an individual’s birth year [28]. Although these reports are important, they do not give insight into the mechanisms of the imprinting event. At this time, we hypothesize that the unique features of the infant immune system may support influenza imprinting by eliciting both broadly reactive responses and long-lived immune memory development of these specific viral antigens. In our next sections, we explore the features of the infant immune system that may contribute to this phenomenon as well as mechanistic studies in preclinical models that may give insight into the imprinting mechanisms.

## 4. Infant Immune Development

**Figure 3:**
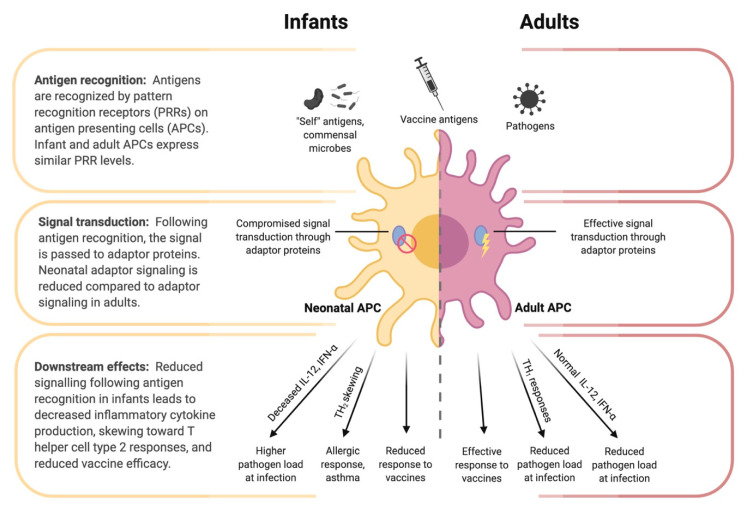
Differences in antigen presenting cell (APC) function between infants and adults. Despite similar pattern recognition receptor (PRR) expression between infant and adult APCs, the downstream signaling following antigen recognition by PRRs differs. Reduced adaptor protein function (e.g., IRF-4) in neonatal APCs contributes to tolerance of self-antigens and commensal microbes, while also reducing responsiveness to pathogens and vaccine antigens, decreased inflammatory cytokine production, and skewing toward a T helper cell type 2 response.

To understand the imprinting event and how it can be applied to optimize infant influenza vaccination, we must first consider the unique features of the early immune system. These features, along with the still-developing respiratory tract, provide the physiological context in which the imprinting event occurs. The greatest challenge facing the neonatal immune system is the transition from the relatively sterile intrauterine environment to a world of tremendous antigenic variation. During this transition, infants have two seemingly contradictory challenges: they must learn to co-exist with commensal microbes, but also learn to eliminate invasive pathogens [55]. Neonates are known to be particularly susceptible to infection for a number of possible reasons. One reason may be the lack of immunological memory that is necessary to mount efficient immune responses to specific antigens [11]. Another reason may be that infants have less specific immune cell subsets compared to adults, which has been shown in mice, although this should not be mistaken for an immune deficiency [11,56]. Finally, some evidence suggests that neonatal immune cells are qualitatively different from adults; immune cell subtypes are not only present in different proportions, but there are phenotypic differences as well [8]. Neonatal immune systems are often described as immature. Indeed, in vitro and in vivo studies have shown some deficiency and immune deviation among infant B cells, T cells, and antigen presenting cells, see Figure 3 [11]. However, three studies in 1996 showed that infant mice were capable of mature T-cell responses under the right circumstances [57,58,59]. It is thus important to distinguish the unique features of the infant immune system from immaturity or immunodeficiency.

### 4.1. Passive Immunity through Maternal Antibodies

Early immunity against specific antigens is partially conferred by maternal immunoglobulins (Ig). During the third trimester of pregnancy, significant amounts of maternal IgG are actively transported across the placenta [60]. This protection is short-lived, as maternal IgG levels in the infant decrease over the first few months of life [61]. Breast-fed infants also obtain maternal IgA produced by mammary gland lymphocytes post-partum [60]. Influenza infection is also typically more frequent in infants 6 to 12 months old compared to newborns to 6 months, which is partially because of waning maternal antibody [62]. Randomized controlled clinical trials have demonstrated that influenza vaccination during pregnancy can reduce the incidence of influenza in offspring. In addition, infants born to influenza-vaccinated mothers have significantly increased hemagglutinin inhibition antibody titers at birth and 2–3 months of age compared to those born to unvaccinated mothers [63].

### 4.2. Innate Immunity of the Infant

There are some major similarities and differences between adult and infant innate immune system components. In response to infection, neonates can produce interleukin (IL)-6 at the same or greater levels compared to adults [64,65]. During respiratory infection in premature and newborn infants, the antimicrobial peptides human beta-defensin 1, human beta-defensin 2 and the cathelicidin LL-37/hCAP-18 are already present and significantly increased in the lungs [66]. Levels of these peptides were correlated with each other and with levels of IL-8 and TNF-α [66]. Unfortunately, newborns are deficient in other soluble immune factors such as components of the complement system, particularly in pre-term and low birth weight infants [8,9]. As newborns age, whole complement activity and components of the classical (C1q, C4, C3) and alternative pathway (factor B, properdin) increase significantly [67]. In addition, neonatal neutrophils are present in lower numbers and lacking in functionality. Neonatal antigen-presenting cells, such as macrophages and dendritic cells, show decreased expression of co-stimulatory molecules, major histocompatibility complex (MHC)-II, and toll-like receptors (TLR) [10]. Innate lymphoid cells (ILCs) may have a compensatory role during this time; ILCs appear and are programmed long before birth, during the embryonic stage [68,69,70]. ILCs show higher levels of activity in infants compared to adults [10].

### 4.3. Adaptive Immune Development in Early Life

As early as 14-weeks gestation, mature B and T cells with a remarkable range of antigenic diversity are already circulating, although there is relatively little antigen present [71]. Infants are capable of both cell-mediated and humoral immunity at birth. This includes production of all Ig isotypes, development of Th1 and Th2 subsets, and cytotoxic T cell responses [72,73]. Hours after birth, colonization of the infant gastrointestinal tract begins [74] and within a week, microbe-specific IgA produced in the intestine can be detected [75].

Infants were once believed to be immunodeficient because of limited production of IL-2; however, the distinction between Th1 and Th2 T helper cell subsets showed that infant immune responses simply have increased Th2 lineage cells [11]. This profile of infant immune response is associated with decreased cell-mediated immunity and may be due to ineffective activation of the innate immune system in early life. Infants have decreased Toll-like receptor (TLR) responses which leads to skewed downstream cytokine production. For example, after TLR stimulation, infant immune cells have decreased production of Th1 cytokines such as interferon (IFN)-α and IFN-γ, and increased Th2 cytokines such as IL-10 [76,77].

Infant B cell responses are considered to be deficient compared to older children and adults—they do not respond fully to T cell-independent antigens until about two years of age [12]. Mutated IgM and IgD B cells undergo somatic hypermutation at the rate of adults by age two [13] while IgG and IgA subtypes only acquire mutations at 60–75% of adult frequencies by age three [13]. The costimulatory receptors CD40, CD80 and CD86 are expressed in lower levels on neonatal naïve B cells compared to adult B cells [78]. In combination with immature B cell, dendritic cell and T cell interactions, B cell activation is limited [78]. This can affect the levels of activated B cells that proliferate, undergo somatic hypermutation, undergo affinity maturation and switch from IgM to IgG, IgA and IgE producing cells [78]. In addition, infant germinal center B cells favor the induction of memory B-cell responses over antibody-secreting plasma cells [78]. The reduction in plasma cells results in lower peak IgG titers. Specifically, plasma B cells exhibit limited IgG response to protein antigen in infants under 12 months of age and polysaccharide antigens for infants under 18–24 months of age [78].

## 5. Infant Immune Responses to Vaccination

Ideally better understanding of infant immune responses to vaccination compared to those of adults could lead to the development of improved infant-specific vaccines.

As previously stated, maternal influenza vaccination is shown to decrease the risk of influenza infection in infants up to 6-months post-partum, and protection does not seem to differ significantly with the timing of maternal vaccination [79]. At birth, infants are capable of protective immune responses to vaccination [73]. The ratio of IgG2 to IgG1 in infants is lower when compared to adults [73]. Antibody responses before 12 months of age are usually lower in durability than those in older persons [73]. It has also been shown that there is an age-dependent increase in the vaccination induced seroconversion and antibody concentration in infants after vaccination [73]. There is an age-dependent limitation of infant antibody responses, and adult levels of protective IgG and IgA are only reached at 12 months or later [73].

Most influenza vaccines elicit suboptimal responses in young children. IIV and subunit vaccines have limited efficacy in infants [71]. Antigen-presenting cells and T cells have lower specific activity during infancy which may also influence responses to influenza vaccines [73]. The addition of adjuvants such as MF59 in FLUAD^TM^ pediatric has been shown to increase vaccine responses in children [80]. Additionally, the LAIV platform has been shown to be most effective in the younger age groups [81]. This may possibly be due to the live viruses present in the vaccine formulation as well as the presentation directly to the respiratory tract [82]. More work needs to be done to determine if vaccination in the young with LAIV can also lead to imprinting.

Vaccination in infants does not elicit the same level of protective immune response as vaccination in adults. It will be important to consider possible mechanistic differences that lead to the different vaccination response, and to do so, proper animal models for infant immune studies must be established.

## 6. Preclinical Modeling of Influenza Virus Infection and Vaccination

Experimental animal studies have been the cornerstone for biological discoveries. We have limited ability to conduct ethical and controlled experiments in humans, and even less ability in infant humans. The respiratory tract and the immune system of infants are significantly different from those of the adults; these differences are the probable causes of variable responses during infection and vaccination [83]. To explore the differences between adult and infant immune responses during influenza virus infection, researchers have often turned to the use of infant animal models to gain insight into the severe respiratory disease of infants. With the use of infant animals, the nuances of adaptive immune regulation such as the imbalance of Th2 responses can be discovered through blood and tissue collection and subsequent leukocyte population and immune mediator analysis. Below, we review the work of investigators using infant animal models such as the mouse and ferret for influenza virus infection studies and highlight the experimental specifics. It is important to note that gaps still exist in development of these models as well as in the studies that have been performed. Most of the investigations have revolved around the mechanisms of cell mediated immunity as it perhaps is easier to study. In this section we explore how preclinical animal models could improve our understanding of infant immune imprinting and the development of influenza vaccines.

### 6.1. Mouse Models

While the use of infant animals for influenza virus infection and vaccination research has not been standardized, mouse models are the main experimental animal for other immune studies. Mice are preferred due to their small size, relatively low main maintenance cost, and ability to reproduce quickly [84]. The gestation of a pregnant mouse is typically 21 days, and after birth neonatal and young mice are milk fed from their mothers until weaning at 3–4 weeks old [85]. Similarly to humans, mice must undergo additional development of the immune system and respiratory system after birth since they are born with some limitations [86]. For example, at birth, T cells in humans and rodents have reduced ability to help immunoglobulin production due to reduction in IL-2, IL-4, and INF-gamma secretion [86]. In respect to B cells in humans and rodents, both heavy and light chain rearrangement has been shown to be fully intact in the fetal spleen but with reduced expression of lymphopoiesis enzymes [86]. Notably, circulating IgM concentrations are reduced to 10% of that of the adult reaching adult levels of IgM at 2 years and IgG at 4–6 years in humans [86]. Although there are many similarities between human and mouse immune systems, there are also differences that should be taken into consideration when designing infant mouse experiments. For example, unlike humans, it has been noted that mice have very reduced lymphocyte levels at birth [87,88] and the T cells seem to have a memory phenotype. Another element that must be considered is the corresponding ages of infant mice and infant humans. The 3-day-old mice neonates are considered representative of late term human neonates of 22–26 weeks gestation [89,90]. Therefore, many infant influenza studies using the mouse model have studied mice in the neonatal phase 2–7 days post-birth which is actually modeling humans in the second trimester of development [90,91,92,93,94]. It has also been reported that the mouse immune makeup at two weeks postpartum most resembles that of the infant human [95], and thus many other infant mice model studies of influenza virus infection have been done at two weeks postpartum [95].

With this in consideration, the use of neonatal and infant mice has been instrumental in learning about the infant immune responses post influenza virus infection. Firstly, results have suggested that neonatal/infant mice are able to clear low levels of viral infection by immune responses regulated differently than compared to adult mice. Specifically, γδ T cells have been shown to protect neonatal mice against mortality following influenza virus infection [91]. In one study, wild-type 7-day old mice infected with A/HKx31 (H3N2) influenza virus recovered from intranasal infection, while γδ T cell deficient neonatal mice had a greater percentage succumb to infection [91]. Further investigation suggested that the γδ T cell protective role is due to IL-17A secretion, which contributes to the infiltration of group 2 innate lymphoid cells and regulatory T cells that can promote lung repair during neonatal influenza virus infection [91]. Another study suggested that infant mice, 2 weeks postpartum, had decreased ability to generate tissue-resident memory (TRM) T cells following influenza virus infection despite a robust CD4+ and CD8+ respiratory localized response for virus clearance [95]. This is interesting because adult mice are able to elicit lung-localized TRM T cells following respiratory virus infection or vaccination, which allows for a rapid response to secondary challenge [96,97]. Another study observed differences in the migration of T cells into the murine respiratory tract following influenza virus infection in infants compared to adults [92]. In this study, mice were infected at 2-days-old, differing from the 2-week-old mice in the previous study. Despite the difference in mouse ages, the young mice had decreased ability to establish TRM T cells due to decreased access of T cells to the lung alveoli. Instead of infiltrating the alveoli, the T cells remained in the interstitium appearing to have dysfunctional migration. The lack of migration of T cells into the airways in infants may possibly be due to differential regulation of CXCR3 ligands CXCL9 and CCL2, which were detected in the adult lung but not in the neonatal lung [92]. Neonatal mice infected with influenza virus have also been shown to have increased expression of CD31 on T cells, which was shown to inhibit T cell activation in the lung when compared to adult mice infected with influenza virus [93]. These studies may have shed some light on the mechanisms of influenza virus imprinting. Follow up on why infant mice have an absence of localized memory responses should be viewed in relation to influenza imprinting and the establishment of a broader peripheral immune memory.

In addition to the above work, studies have shown that neonates have an increased ability to develop inducible Bronchus Associated Lymphoid Tissue (iBALT) within the lungs [17]. iBALT is an ectopic tertiary lymphoid tissue that forms in the lung following respiratory insult and serves as an area for local antigen capture and T and B cell stimulation [22]. It is composed of follicular dendritic cells, resident dendritic cells, high endothelial venules and lymphocytes, and has previously been shown to be established in infants during respiratory infection. A study using neonatal mice showed that IL-17A producing T cells were essential for iBALT establishment in the neonatal lung after antigen exposure [17]. Interestingly, the number of T cells and B cells in the alveolar space were decreased in neonatal mice and the lymphocytes were organized in the iBALT structure in the alveoli [17].

Looking at these studies together, there is accumulating evidence to suggest that there is decreased activity, recruitment, and memory establishment of T lymphocytes in infants following influenza virus infection in the mouse model. These studies show how the infant regulates the immune response to prevent an overactive filtration of T cells into the delicate immature lung. We first recognize from this review of the literature that there are few studies investigating the humoral response and antibody evolution following influenza virus infection in the infant mouse model. Furthermore, we see a trend of decreased T cell immune responses as expected from what is understood of the Th2 polarization of the infant immune system. It is also evident that there is decreased recruitment of T cells into the alveolar space, which may be a clue to the establishment of influenza imprinting.

### 6.2. Ferret Model

The ferret model has also been used to investigate age-related host response and disease severity to influenza virus infection both for the old [98] and young [19,20,99,100]. Ferrets were first used to investigate infant responses to influenza virus infection in the late 1970s by a group from the University of Birmingham in the United Kingdom [21,101,102,103,104,105]. In adult ferrets, influenza virus infection with seasonal influenza virus strains typically leads to a non-fatal disease characterized by weight loss, temperature increase, and respiratory symptoms including coughing and sneezing [106,107] at varying degrees dependent on the strain. In infant ferrets, studies have shown a trend that suggests that influenza disease severity is dependent on ferret age throughout immaturity prior to adulthood. As we examine the following studies using the ferret model, we will see how this model has contributed to a better understanding of the infant response to influenza virus infection.

A study by Collie and colleagues found that seasonal influenza virus infection in newborn ferrets with a recombinant H3N2 virus strain led to mortality, significant viral replication within the respiratory tract, and evidence of severe respiratory pathology including collapsed alveolar spaces, necrotizing bronchiolitis, and death [105]. In a follow up study that compared influenza illness in newborn ferrets to 15-day-old suckling ferrets, it was found that while newborn ferrets succumb to illness, 15-day old ferrets seemed resilient to fatal disease and developed pathology similar to adults [108]. Furthermore, the increased disease severity in infants was suggested to be due to an increased proportion of ciliated epithelium-lined airway when compared to the adult and 15-day-ferret lungs [21]. This suggested a rapid development of the ferret respiratory tract, which may influence influenza severity. In our studies, using infant (4-week-old) and newly-weaned (8-week-old) ferrets inoculated with the 2009 H1N1 pandemic virus, it was found that infant ferrets had a 100% mortality rate while the 8-week-old newly weaned ferrets did not display any significant clinical symptoms [19,20]. Pathological and virological assessments of the respiratory tracts indicated similar levels of replicating virus but the infant ferret lungs had evidence of pathology with significant T cell infiltration into the submucosal glands [19]. Interestingly, the newly weaned animals had minimal signs of leukocyte infiltration into the respiratory tract. Clear alveolar spaces were noted with structures surrounding the bronchi similar to iBALT with organized T cell and B cell zones. This organization has also been observed in infant respiratory infection and insult [20,22]. Similar results were found in another study inoculating the newly weaned age group with pandemic H1N1 influenza A virus strains [100]. Taken together, these studies suggest that due to the respiratory tract development in ferrets post-partum, the outcome of influenza virus infection may be significantly dependent on the age of the ferret by week postpartum. It is known that the ferret respiratory tract is not fully developed until at least 8 weeks of age which coincides with weaning. Considering the direct susceptibility of the ferret to human strains of influenza viruses, the newborn, infant, and newly weaned ferret may serve as an appropriate model for determining strain and age-specific outcomes of new influenza virus strains and the understanding of imprinting mechanisms [109].

### 6.3. Non-Human Primates

Non-human primates (NHPs) have been effective models of influenza virus infection and transmission for over a century [110]. Several NHP species are shown to be susceptible to infection with certain human influenza viruses. For example, Moncla and colleagues found that the common marmoset was susceptible to infection with non-adapted H1N1pdm09, with infected animals seroconverting and displaying human-like symptoms [111]. Cynomolgus macaques are highly susceptible to avian H5N1 which can infect humans [112,113], and moderately susceptible to human H3N2 [114] and H1N1 [113].

Rhesus macaques (RMs) may be an effective model of infant immunity, as their immune ontogeny throughout fetal development and following birth is incredibly similar to that of human infants [115]. However, a study by Merino and colleagues reported that as of 2020, no studies showed comprehensive data on the clinical chemistry or hematology of pediatric rhesus macaques in early development [116]. Therefore, they aimed to generate this reference data for pediatric RMs themselves, and showed that RMs in early life share several immune landmarks with human infants. First, they showed that the number of B cells, CD1c+ DCs, CD4+ T cells, and CD8+ T cells increase substantially over the first six months of life, and that these counts were generally increased in colony-reared macaques compared research animals [116]. Additionally, they showed that pediatric RMs express higher levels of IL-13, IL-10, and TNFα compared to adults, while expressing lowered levels of proinflammatory cytokines IL-1β and IL-12. Like human infants, this cytokine profile in the pediatric RM was represented by Th2 immunity and antigen tolerance. Another study showed that RMs follow similar age-related trends in immunity as humans, such as changes in the frequency and function and peripheral blood lymphocytes throughout the lifespan [117].

Despite these important similarities between the RM and human infant, macaques are generally not optimal models of human influenza virus infection [118]. Where ferrets and pigs showed similar patterns of viral attachment (PVAs) to humans when infected with H1N1 and H3N2, such as binding to ciliated epithelial cells in the airway and to type 1 pneumocytes in alveoli, macaques had very dissimilar PVAs [118]. Notably, H1N1 and H3N2 viral attachment was not observed in the respiratory tracts of RMs, and only moderate viral attachment to the bronchial epithelia was observed for H5N1, H5N9, and H6N1 [118]. Other studies have shown successful H1N1 and H3N2 infection in cynomolgus and rhesus macaques, with variable clinical signs [114,119,120,121,122]. Nonetheless, an important limitation to NHP infant models is the susceptibility to human influenza viruses, as the optimal infant model is not necessarily the optimal model of viral infection. The benefit of NHPs would be the large complement of reagents that would allow immune dissection and mechanistic study.

When considering vaccination studies, some investigations have applied non-human primate models to study influenza virus infection and vaccination strategies in infants. For example, a study by Kim and colleagues using 4–6-day-old African green monkeys (AGMs) showed that vaccines containing flagellin, a TLR5 agonist, enhanced IgG responses at following vaccination boost and challenge [123]. Another study by Holbrook et al. using infant AGMs showed higher viral load in the lungs, reduced influenza-specific IgG in the lungs, and impaired formation of BALT following influenza virus infection [124]. They also validated the findings of enhanced immune responses in neonatal AGMs to flagellin-adjuvanted inactivated influenza vaccines [125], and later showed that R848 was more effective than flagellin at enhancing B cell activation in draining lymph nodes post-vaccination [126].

The purpose of this section was to highlight the importance of using appropriate animal models for influenza virus infection studies. After reviewing studies conducted in both infant mice, ferrets, and non-human primates, it is clear that these models are able to provide valuable information regarding the infant immune system, but more development needs to be done. We need an infant animal model that shares the unique immune features of a human infant in order to understand the influenza virus imprinting event so we can design vaccines that better cater to the infant immune response.

## 7. Can We Use the Knowledge of the Infant Immune System and Influenza Imprinting to Design Better Vaccines?

In this review of the infant immune system and influenza, we understand that there are unique features of immune regulation and pathogen response in infants; that influenza infection during early life can lead to life-long immunity (imprinting), and that the influenza vaccine is poorly immunogenic in the younger age groups. Synthesizing these bits of knowledge, we ask two questions: (1) Can understanding the infant immune interaction with the influenza virus identify mechanisms that lead to immune imprinting and broader immune responses toward influenza viral antigens? and (2) Can we utilize imprinting knowledge to develop better influenza virus vaccines? When considering our first question, there has been much effort placed into how broadly reactive immune responses can be employed for universal influenza vaccine development [127]. Specifically, interest has revolved around leveraging conserved epitopes of the HA stem or of more conserved proteins such as the nucleoprotein NP to elicit protective responses toward a more diverse group of viruses [128,129,130].

We note that these strategies are mostly concentrated on the virus while there have been fewer studies focusing on manipulating the immune system for broader responses [131]. Understanding how the immune system can be leveraged for a broader immune response will help move vaccine development forward. Imprinting has been associated with broader and long-lived immunity, however, the specific cellular events surrounding this imprinting event are ill-defined. What we do know from examination of the infant immune system is that infants have differential rates of somatic hypermutation, lower levels of particular soluble immune factors, favor memory B cell establishment, and have lower levels of costimulatory molecules. It seems highly possible that these features are key to infant viral imprinting. In particular, influenza imprinting has been speculated to establish a broadly reactive immune response, which can be recalled against antigenically distinct strains in the future [47]. Given that the rate of infant BCR somatic hypermutation is decreased compared to that of adults [13], the specificity of BCRs for influenza virus antigen could theoretically be lower compared to BCR specificity in an adult, which could lead to a broader response in infants. Perhaps, lower affinity memory B cells established in infancy are key contributors to the imprinting effect and protection later in life. Moreover, lower levels off co-stimulatory molecules may also lead to more broader responses. More work is needed to dissect the roles of these immune mechanisms in influenza imprinting. To do this, more animal studies that utilize infant animals to investigate the immune memory developed toward influenza virus antigens are needed. Very few studies at this time use infant animals and even fewer follow infected or vaccinated subjects throughout development. Our review of the literature suggests that mice, ferrets, and non-human primates all have benefits as well as drawbacks as models for study of infant influenza studies and defining the mechanisms of imprinting. Studies from mice suggest that iBALT is an important immune structure developed in infants which serve for local immune education and development for a fast response to insult. Further investigation of the respiratory tract of infants should be conducted to determine how iBALT may be involved in imprinting.

When considering our second question—Can we utilize imprinting knowledge to develop better influenza virus vaccines?—we must first recognize that there are two major issues with in respect to influenza vaccines. This first is that infants have decreased immune responses to influenza vaccine immunizations. The second is that the current seasonal vaccine is directed toward specific antigens of circulating viruses which necessitates vaccine updating each year. In this review we wanted to determine if understanding infant immunity and influenza virus infection could offer any insight for future vaccine design. The addition of adjuvants to the current vaccine formulations has been successful in clinical trials of MF-59-adjuvanted influenza vaccine. As well, we know that infants have decreased toll-like receptors (TLRs) which may significantly affect the stimulation of inflammation for antigen up-take at vaccination. It is possible that tailoring adjuvants toward the unique expression pattern of TLRs during infancy and childhood may be an effective strategy for increasing immunization effectiveness specifically of influenza vaccines. Moreover, using vaccines that can elicit iBALT or possibly NALT (Nasal Associated Lymphoid Tissue) in infants and adults may be an effective strategy for the development of a local and possible broader immune response. Considering this, work is needed to determine if local immune structures such as iBALT and NALT can induce broader responses. However, since we do know that the LAIV is more effective in young children, investigation into the memory induction after LAIV vaccination may give insight into the use of LAIV as an imprinting vaccine. With regard to adult vaccination strategies, identifying the mechanisms that the infant immune system uses to decrease somatic hypermutation and increase B cell memory induction may provide clues to how we can stimulate these pathways during adult vaccination for a broader and more long-lived immune response at influenza vaccination. Moving forward, comprehensive studies of influenza vaccination in infancy and adulthood in both preclinical animal models should examine the type of antigen, adjuvants, route of vaccination, vaccination schedule (e.g., single dose versus boost), and host age that maximizes the specificity and long-lived memory of the immune response.

## 8. Concluding Statements

Infant immune systems and respiratory tracts differ significantly from those of adults. Infants and young children have increased rates of influenza virus infection, hospitalization, and mortality compared to older persons. Due to some of these immune differences and insufficient data on infant influenza vaccination, currently available vaccines cannot be used in infants less than 6 months, and others used in older infants result in dampened immune responses in infants, providing inadequate protection to future influenza virus infection. We note the importance of the imprinting event and how this significant event can shape the immune response to future infections. Currently there is no standardized animal model for infant influenza and immune research. Both mice and ferrets have been used in these previous studies. There are some gaps as these preclinical studies primarily examine cell-mediated immunity rather than humoral immunity and many of the studies are focused on infection rather than vaccination in infants. In order to move forward, studies investigating infant immune response to vaccination should be conducted in infant animal models of appropriate age as well as in humans. Understanding how vaccination can serve as the imprinting event will inform the development of more efficacious vaccines and better infant vaccination policies. This is necessary work if we wish to keep infants as safe as possible during seasonal influenza epidemics and may also provide the keys to developing a broadly reactive and long-lived universal influenza vaccine that will continue to provide protection into adulthood.

## Figures and Tables

**Table 1:** Available influenza vaccine platforms by age group. [34,39,40].

Age Group	Recommended Vaccine	Live Vaccine (Yes/No)	Reason for Recommendation
**Infants aged 0–6 months**	None; maternal vaccination during pregnancy recommended	No	No licensed influenza vaccines for infants less than 6 months of age
**Infants aged 6 months–2 years**	TIV or QIV	No	LAIV is not authorized for use in children less than two years of age
**Children aged 2–17 years**	TIV, QIV or LAIV	Yes	All vaccines authorized for age group
**Adults aged 18–59 years**	TIV, QIV or LAIV	Yes	All vaccines authorized for age group
**Adults 60–64 years**	TIV and QIV	No	LAIV not recommended
**Adults aged 65 and over**	Adjuvanted TIV or high dose TIV	No	Unadjuvanted vaccines are poorly immunogenic in elderly populations

TIV: trivalent influenza vaccine; QIV: quadrivalent influenza vaccine; LAIV: live attenuated influenza vaccine.

## References

[1] Krammer F., Smith G.J.D., Fouchier R.A.M., Peiris M., Kedzierska K., Doherty P.C., Palese P., Shaw M.L., Treanor J., Webster R.G. (2018). Influenza. Nat. Rev. Dis. Primers.

[2] Kim H., Webster R.G., Webby R.J. (2018). Influenza virus: Dealing with a drifting and shifting pathogen. Viral. Immunol..

[3] WHO|Influenza. http://www.who.int/biologicals/vaccines/influenza/en/.

[4] Jane M., Vidal M.J., Soldevila N., Romero A., Martinez A., Torner N., Godoy P., Launes C., Rius C., Marcos M.A. (2019). Epidemiological and clinical characteristics of children hospitalized due to influenza A and B in the south of Europe, 2010–2016. Sci. Rep..

[5] Nair H., Brooks W.A., Katz M., Roca A., Berkley J.A., Madhi S.A., Simmerman J.M., Gordon A., Sato M., Howie S. (2011). Global burden of respiratory infections due to seasonal influenza in young children: A systematic review and meta-analysis. Lancet.

[6] Neuzil K.M., Mellen B.G., Wright P.F., Mitchel E.F., Griffin M.R. (2000). The effect of influenza on hospitalizations, outpatient visits, and courses of antibiotics in children. N. Engl. J. Med..

[7] Poehling K.A., Edwards K.M., Weinberg G.A., Szilagyi P., Staat M.A., Iwane M.K., Bridges C.B., Grijalva C.G., Zhu Y., Bernstein D.I. (2006). The underrecognized burden of influenza in young children. N. Engl. J. Med..

[8] Adkins B. (2013). Neonatal immunology: Responses to pathogenic microorganisms and epigenetics reveal an “immunodiverse” developmental state. Immunol. Res..

[9] Krediet T.G., Beurskens F.J., van Dijk H., Gerards L.J., Fleer A. (1998). Antibody responses and opsonic activity in sera of preterm neonates with coagulase-negative staphylococcal septicemia and the effect of the administration of fresh frozen plasma. Pediatr. Res..

[10] Yu J.C., Khodadadi H., Malik A., Davidson B., Salles E., Bhatia J., Hale V.L., Baban B. (2018). Innate immunity of neonates and infants. Front. Immunol..

[11] Adkins B., Leclerc C., Marshall-Clarke S. (2004). Neonatal adaptive immunity comes of age. Nat. Rev. Immunol..

[12] Rijkers G.T., Dollekamp E.G., Zegers B.J. (1987). The in vitro B-cell response to pneumococcal polysaccharides in adults and neonates. Scand. J. Immunol..

[13] Nielsen S.C.A., Roskin K.M., Jackson K.J.L., Joshi S.A., Nejad P., Lee J.Y., Wagar L.E., Pham T.D., Hoh R.A., Nguyen K.D. (2019). Shaping of infant B cell receptor repertoires by environmental factors and infectious disease. Sci. Transl. Med..

[14] Mazagatos C., Godoy P., Muñoz Almagro C., Pozo F., Larrauri A. (2020). Effectiveness of influenza vaccination during pregnancy to prevent severe infection in children under 6 months of age, Spain, 2017–2019. Vaccine.

[15] Grohskopf L.A., Alyanak E., Broder K.R., Walter E.B., Fry A.M., Jernigan D.B. (2019). Prevention and control of seasonal influenza with vaccines: Recommendations of the advisory committee on immunization practices—United States, 2019–2020 Influenza Season. MMWR Recomm. Rep..

[16] Kelvin A.A., Zambon M. (2019). Influenza imprinting in childhood and the influence on vaccine response later in life. Euro. Surveill..

[17] Rangel-Moreno J., Carragher D.M., de la Luz Garcia-Hernandez M., Hwang J.Y., Kusser K., Hartson L., Kolls J.K., Khader S.A., Randall T.D. (2012). Induction of BALT in the absence of IL-17. Nat. Immunol..

[18] Verhoeven D., Perry S., Pryharski K. (2016). Control of influenza infection is impaired by diminished interferon-γ secretion by CD4 T cells in the lungs of toddler mice. J. Leukoc. Biol..

[19] Paquette S.G., Banner D., Huang S.S.H., Almansa R., Leon A., Xu L., Bartoszko J., Kelvin D.J., Kelvin A.A. (2015). Influenza transmission in the mother-infant dyad leads to severe disease, mammary gland infection, and pathogenesis by regulating host responses. PLoS Pathog..

[20] Huang S.S.H., Banner D., Degousee N., Leon A.J., Xu L., Paquette S.G., Kanagasabai T., Fang Y., Rubino S., Rubin B. (2012). Differential pathological and immune responses in newly weaned ferrets are associated with a mild clinical outcome of pandemic 2009 H1N1 infection. J. Virol..

[21] Coates D.M., Husseini R.H., Rushton D.I., Sweet C., Smith H. (1984). The role of lung development in the age-related susceptibility of ferrets to influenza virus. Br. J. Exp. Pathol..

[22] Rangel-Moreno J., Hartson L., Navarro C., Gaxiola M., Selman M., Randall T.D. (2006). Inducible bronchus-associated lymphoid tissue (iBALT) in patients with pulmonary complications of rheumatoid arthritis. J. Clin. Investig..

[23] Bouvier N.M., Palese P. (2008). The biology of influenza viruses. Vaccine.

[24] Influenza (Seasonal). https://www.who.int/news-room/fact-sheets/detail/influenza-.

[25] Jang J., Bae S.E. (2018). Comparative co-evolution analysis between the HA and NA genes of influenza A virus. Virology (Auckl).

[26] Gamblin S.J., Skehel J.J. (2010). Influenza hemagglutinin and neuraminidase membrane glycoproteins. J. Biol. Chem..

[27] Chen C.-J., Ermler M.E., Tan G.S., Krammer F., Palese P., Hai R. (2016). Influenza A viruses expressing intra- or intergroup chimeric hemagglutinins. J. Virol..

[28] Francis M.E., King M.L., Kelvin A.A. (2019). Back to the future for influenza preimmunity-looking back at influenza virus history to infer the outcome of future infections. Viruses.

[29] Kalil A.C., Thomas P.G. (2019). Influenza virus-related critical illness: Pathophysiology and epidemiology. Crit. Care.

[30] Taubenberger J.K., Morens D.M. (2008). The pathology of influenza virus infections. Annu. Rev. Pathol..

[31] Garg S., Jain S., Dawood F.S., Jhung M., Pérez A., D’Mello T., Reingold A., Gershman K., Meek J., Arnold K.E. (2015). Pneumonia among adults hospitalized with laboratory-confirmed seasonal influenza virus infection—United States, 2005–2008. BMC Infect. Dis..

[32] Mauad T., Hajjar L.A., Callegari G.D., da Silva L.F.F., Schout D., Galas F.R.B.G., Alves V.A.F., Malheiros D.M.A.C., Auler J.O.C., Ferreira A.F. (2010). Lung pathology in fatal novel human influenza A (H1N1) infection. Am. J. Respir. Crit. Care Med..

[33] Vemula S.V., Sayedahmed E.E., Sambhara S., Mittal S.K. (2017). Vaccine approaches conferring cross-protection against influenza viruses. Expert Rev. Vaccines.

[34] Fiore A.E., Shay D.K., Broder K., Iskander J.K., Uyeki T.M., Mootrey G., Bresee J.S., Cox N.J., Centers for Disease Control and Prevention (2009). Prevention and control of seasonal influenza with vaccines: Recommendations of the Advisory Committee on Immunization Practices (ACIP), 2009. MMWR Recomm. Rep..

[35] Wong S.-S., Webby R.J. (2013). Traditional and new influenza vaccines. Clin. Microbiol. Rev..

[36] Rajao D.S., Perez D.R. (2018). Universal vaccines and vaccine platforms to protect against influenza viruses in humans and agriculture. Front. Microbiol..

[37] Adjuvanted Influenza Vaccines. https://www.ncbi.nlm.nih.gov/pmc/articles/PMC5861793/.

[38] Del Giudice G., Rappuoli R., Oldstone M.B.A., Compans R.W. (2015). Inactivated and adjuvanted influenza vaccines. Influenza Pathogenesis and Control—Volume II.

[39] Public Health Agency of Canada. Canadian Immunization Guide Chapter on Influenza and Statement on Seasonal Influenza Vaccine for 2019–2020.

[40] Influenza Vaccination: A Summary for Clinicians|CDC. https://www.cdc.gov/flu/professionals/vaccination/vax-summary.htm.

[41] Hancock K., Veguilla V., Lu X., Zhong W., Butler E.N., Sun H., Liu F., Dong L., DeVos J.R., Gargiullo P.M. (2009). Cross-reactive antibody responses to the 2009 pandemic H1N1 influenza virus. N. Engl. J. Med..

[42] Francis T. (1960). On the doctrine of original antigenic sin. Proc. Am. Philos. Soc..

[43] Fonville J.M., Wilks S.H., James S.L., Fox A., Ventresca M., Aban M., Xue L., Jones T.C., Le N.M.H., Pham Q.T. (2014). Antibody landscapes after influenza virus infection or vaccination. Science.

[44] Monto A.S., Malosh R.E., Petrie J.G., Martin E.T. (2017). The doctrine of original antigenic sin: Separating good from evil. J. Infect. Dis..

[45] Lessler J., Riley S., Read J.M., Wang S., Zhu H., Smith G.J.D., Guan Y., Jiang C.Q., Cummings D.A.T. (2012). Evidence for antigenic seniority in influenza A (H3N2) antibody responses in southern China. PLoS Pathog..

[46] Skowronski D.M., Chambers C., De Serres G., Sabaiduc S., Winter A.L., Dickinson J.A., Gubbay J.B., Fonseca K., Drews S.J., Charest H. (2017). Serial vaccination and the antigenic distance hypothesis: Effects on influenza vaccine effectiveness during A(H3N2) epidemics in Canada, 2010–2011 to 2014–2015. J. Infect. Dis..

[47] Gostic K.M., Ambrose M., Worobey M., Lloyd-Smith J.O. (2016). Potent protection against H5N1 and H7N9 influenza via childhood hemagglutinin imprinting. Science.

[48] Andrews S.F., Huang Y., Kaur K., Popova L.I., Ho I.Y., Pauli N.T., Henry Dunand C.J., Taylor W.M., Lim S., Huang M. (2015). Immune history profoundly affects broadly protective B cell responses to influenza. Sci. Transl. Med..

[49] Ellebedy A.H., Ahmed R. (2012). Re-engaging cross-reactive memory B cells: The influenza puzzle. Front. Immunol..

[50] Worobey M., Han G.-Z., Rambaut A. (2014). Genesis and pathogenesis of the 1918 pandemic H1N1 influenza A virus. Proc. Natl. Acad. Sci. USA.

[51] Arevalo C.P., Le Sage V., Bolton M.J., Eilola T., Jones J.E., Kormuth K.A., Nturibi E., Balmaseda A., Gordon A., Lakdawala S.S. (2020). Original antigenic sin priming of influenza virus hemagglutinin stalk antibodies. Proc. Natl. Acad. Sci. USA.

[52] Tesini B.L., Kanagaiah P., Wang J., Hahn M., Halliley J.L., Chaves F.A., Nguyen P.Q.T., Nogales A., DeDiego M.L., Anderson C.S. (2019). Broad Hemagglutinin-specific memory b cell expansion by seasonal influenza virus infection reflects early-life imprinting and adaptation to the infecting virus. J. Virol..

[53] Skowronski D.M., Sabaiduc S., Leir S., Rose C., Zou M., Murti M., Dickinson J.A., Olsha R., Gubbay J.B., Croxen M.A. (2019). Paradoxical clade- and age-specific vaccine effectiveness during the 2018/19 influenza A(H3N2) epidemic in Canada: Potential imprint-regulated effect of vaccine (I-REV). Euro. Surveill..

[54] Francis M.E., McNeil M., Dawe N.J., Foley M.K., King M.L., Ross T.M., Kelvin A.A. (2019). Historical H1N1 influenza virus imprinting increases vaccine protection by influencing the activity and sustained production of antibodies elicited at vaccination in ferrets. Vaccines.

[55] Strunk T., Currie A., Richmond P., Simmer K., Burgner D. (2011). Innate immunity in human newborn infants: Prematurity means more than immaturity. J. Matern. Fetal. Neonatal. Med..

[56] Fadel S., Sarzotti M. (2000). Cellular immune responses in neonates. Int. Rev. Immunol..

[57] Sarzotti M., Robbins D.S., Hoffman P.M. (1996). Induction of protective CTL responses in newborn mice by a murine retrovirus. Science.

[58] Ridge J.P., Fuchs E.J., Matzinger P. (1996). Neonatal tolerance revisited: Turning on newborn T cells with dendritic cells. Science.

[59] Forsthuber T., Yip H.C., Lehmann P.V. (1996). Induction of TH1 and TH2 immunity in neonatal mice. Science.

[60] Offit P.A., Quarles J., Gerber M.A., Hackett C.J., Marcuse E.K., Kollman T.R., Gellin B.G., Landry S. (2002). Addressing parents’ concerns: Do multiple vaccines overwhelm or weaken the infant’s immune system?. Pediatrics.

[61] Siegrist C.A., Cordova M., Brandt C., Barrios C., Berney M., Tougne C., Kovarik J., Lambert P.H. (1998). Determinants of infant responses to vaccines in presence of maternal antibodies. Vaccine.

[62] Glezen W.P., Taber L.H., Frank A.L., Gruber W.C., Piedra P.A. (1997). Influenza virus infections in infants. Pediatr. Infect. Dis. J..

[63] Eick A.A., Uyeki T.M., Klimov A., Hall H., Reid R., Santosham M., O’Brien K.L. (2011). Maternal influenza vaccination and effect on influenza virus infection in young infants. Arch. Pediatr. Adolesc. Med..

[64] Yachie A., Takano N., Ohta K., Uehara T., Fujita S., Miyawaki T., Taniguchi N. (1992). Defective production of interleukin-6 in very small premature infants in response to bacterial pathogens. Infect. Immun..

[65] Angelone D.F., Wessels M.R., Coughlin M., Suter E.E., Valentini P., Kalish L.A., Levy O. (2006). Innate immunity of the human newborn is polarized toward a high ratio of IL-6/TNF-alpha production in vitro and in vivo. Pediatr. Res..

[66] Schaller-Bals S., Schulze A., Bals R. (2002). Increased levels of antimicrobial peptides in tracheal aspirates of newborn infants during infection. Am. J. Respir. Crit. Care Med..

[67] Drew J.H., Arroyave C.M. (1980). The complement system of the newborn infant. Biol. Neonate..

[68] Diefenbach A., Colonna M., Romagnani C. (2017). The ILC world revisited. Immunity.

[69] Eberl G., Colonna M., Di Santo J.P., McKenzie A.N. (2015). Innate lymphoid cells. Innate lymphoid cells: A new paradigm in immunology. Science.

[70] Artis D., Spits H. (2015). The biology of innate lymphoid cells. Nature.

[71] Goldblatt D. (1998). Immunisation and the maturation of infant immune responses. Dev. Biol. Stand..

[72] Fadel S.A., Cowell L.G., Cao S., Ozaki D.A., Kepler T.B., Steeber D.A., Sarzotti M. (2006). Neonate-primed CD8+ memory cells rival adult-primed memory cells in antigen-driven expansion and anti-viral protection. Int. Immunol..

[73] Siegrist C.A. (2001). Neonatal and early life vaccinology. Vaccine.

[74] Mackie R.I., Sghir A., Gaskins H.R. (1999). Developmental microbial ecology of the neonatal gastrointestinal tract. Am. J. Clin. Nutr..

[75] Mellander L., Carlsson B., Jalil F., Soderstrom T., Hanson L.A. (1985). Secretory IgA antibody response against Escherichia coli antigens in infants in relation to exposure. J. Pediatr..

[76] Zhang X., Deriaud E., Jiao X., Braun D., Leclerc C., Lo-Man R. (2007). Type I interferons protect neonates from acute inflammation through interleukin 10-producing B cells. J. Exp. Med..

[77] Belderbos M.E., Levy O., Stalpers F., Kimpen J.L., Meyaard L., Bont L. (2012). Neonatal plasma polarizes TLR4-mediated cytokine responses towards low IL-12p70 and high IL-10 production via distinct factors. PLoS ONE.

[78] Siegrist C.-A., Aspinall R. (2009). B-cell responses to vaccination at the extremes of age. Nat. Rev. Immunol..

[79] Katz J., Englund J.A., Steinhoff M.C., Khatry S.K., Shrestha L., Kuypers J., Mullany L.C., Chu H.Y., LeClerq S.C., Kozuki N. (2018). Impact of timing of influenza vaccination in pregnancy on transplacental antibody transfer, influenza incidence, and birth outcomes: A randomized trial in rural nepal. Clin. Infect. Dis..

[80] Vesikari T., Knuf M., Wutzler P., Karvonen A., Kieninger-Baum D., Schmitt H.-J., Baehner F., Borkowski A., Tsai T.F., Clemens R. Oil-in-Water Emulsion Adjuvant with Influenza Vaccine in Young Children. https://www.nejm.org/doi/10.1056/NEJMoa1010331.

[81] Coelingh K., Olajide I.R., MacDonald P., Yogev R. (2015). Efficacy and effectiveness of live attenuated influenza vaccine in school-age children. Expert Rev. Vaccines.

[82] Blanco-Lobo P., Nogales A., Rodríguez L., Martínez-Sobrido L. (2019). Novel approaches for the development of live attenuated influenza vaccines. Viruses.

[83] Restori K.H., Srinivasa B.T., Ward B.J., Fixman E.D. (2018). Neonatal immunity, respiratory virus infections, and the development of asthma. Front. Immunol..

[84] Bryda E.C. (2013). The mighty mouse: The impact of rodents on advances in biomedical research. Mo. Med..

[85] Richter S.H., Kästner N., Loddenkemper D.-H., Kaiser S., Sachser N. (2016). A time to wean? Impact of weaning age on anxiety-like behaviour and stability of behavioural traits in full adulthood. PLoS ONE.

[86] Holladay S.D., Smialowicz R.J. (2000). Development of the murine and human immune system: Differential effects of immunotoxicants depend on time of exposure. Environ. Health Perspect..

[87] Garcia A.M., Fadel S.A., Cao S., Sarzotti M. (2000). T cell immunity in neonates. Immunol. Res..

[88] Campion A.L., Bourgeois C., Lambolez F., Martin B., Léaument S., Dautigny N., Tanchot C., Pénit C., Lucas B. (2002). Naive T cells proliferate strongly in neonatal mice in response to self-peptide/self-MHC complexes. Proc. Natl. Acad. Sci. USA.

[89] Fike A.J., Nguyen L.T., Kumova O.K., Carey A.J. (2017). Characterization of CD31 expression on murine and human neonatal T lymphocytes during development and activation. Pediatr. Res..

[90] Carey A.J., Gracias D.T., Thayer J.L., Boesteanu A.C., Kumova O.K., Mueller Y.M., Hope J.L., Fraietta J.A., van Zessen D.B.H., Katsikis P.D. (2016). Rapid evolution of the CD8+ TCR repertoire in neonatal mice. J. Immunol..

[91] Guo X.-Z.J., Dash P., Crawford J.C., Allen E.K., Zamora A.E., Boyd D.F., Duan S., Bajracharya R., Awad W.A., Apiwattanakul N. (2018). Lung γδ T cells mediate protective responses during neonatal influenza infection that are associated with type 2 immunity. Immunity.

[92] Lines J.L., Hoskins S., Hollifield M., Cauley L.S., Garvy B.A. (2010). The migration of T Cells in response to influenza virus is altered in neonatal mice. J. Immunol..

[93] Fike A.J., Kumova O.K., Tardif V.J., Carey A.J. (2019). Neonatal influenza-specific effector CTLs retain elevated CD31 levels at the site of infection and have decreased IFN-γ production. J. Leukoc. Biol..

[94] Oliphant S., Lines J.L., Hollifield M.L., Garvy B.A. (2015). Regulatory T cells are critical for clearing influenza A Virus in neonatal mice. Viral Immunol..

[95] Zens K.D., Chen J.K., Guyer R.S., Wu F.L., Cvetkovski F., Miron M., Farber D.L. (2017). Reduced generation of lung tissue–resident memory T cells during infancy. J. Exp. Med..

[96] Zens K.D., Chen J.K., Farber D.L. (2016). Vaccine-generated lung tissue–resident memory T cells provide heterosubtypic protection to influenza infection. JCI Insight.

[97] Teijaro J.R., Turner D., Pham Q., Wherry E.J., Lefrançois L., Farber D.L. (2011). Tissue-retentive lung memory CD4 T cells mediate optimal protection to respiratory virus infection. J. Immunol..

[98] Paquette S.G., Huang S.S.H., Banner D., Xu L., Leόn A., Kelvin A.A., Kelvin D.J. (2014). Impaired heterologous immunity in aged ferrets during sequential influenza A H1N1 infection. Virology.

[99] Smith H., Sweet C. (1988). Lessons for human influenza from pathogenicity studies with ferrets. Rev. Infect. Dis..

[100] Bissel S.J., Carter C.E., Wang G., Johnson S.K., Lashua L.P., Kelvin A.A., Wiley C.A., Ghedin E., Ross T.M. (2019). Age-related pathology associated with H1N1 A/California/07/2009 influenza virus infection. Am. J. Pathol..

[101] Jakeman K.J., Smith H., Sweet C. (1989). Mechanism of immunity to influenza: Maternal and passive neonatal protection following immunization of adult ferrets with a live vaccinia-influenza virus haemagglutinin recombinant but not with recombinants containing other influenza virus proteins. J. Gen. Virol..

[102] Husseini R.H., Sweet C., Overton H., Smith H. (1984). Role of maternal immunity in the protection of newborn ferrets against infection with a virulent influenza virus. Immunology.

[103] Husseini R.H., Collie M.H., Rushton D.I., Sweet C., Smith H. (1983). The role of naturally-acquired bacterial infection in influenza-related death in neonatal ferrets. Br. J. Exp. Pathol..

[104] Rushton D.I., Collie M.H., Sweet C., Husseini R.H., Smith H. (1983). The effects of maternal influenzal viraemia in late gestation on the conceptus of the pregnant ferret. J. Pathol..

[105] Collie M.H., Rushton D.I., Sweet C., Smith H. (1980). Studies of influenza virus infection in newborn ferrets. J. Med. Microbiol..

[106] Huang S.S.H., Banner D., Paquette S.G., Leon A.J., Kelvin A.A., Kelvin D.J. (2014). Pathogenic influenza B virus in the ferret model establishes lower respiratory tract infection. J. Gen. Virol..

[107] Huang S.S.H., Banner D., Fang Y., Ng D.C.K., Kanagasabai T., Kelvin D.J., Kelvin A.A. (2011). Comparative analyses of pandemic H1N1 and seasonal H1N1, H3N2, and influenza B infections depict distinct clinical pictures in ferrets. PLoS ONE.

[108] Coates D.M., Husseini R.H., Collie M.H., Sweet C., Smith H. (1984). The role of cellular susceptibility in the declining severity of respiratory influenza of ferrets with age. Br. J. Exp. Pathol..

[109] Skarlupka A.L., Ross T.M. (2020). Immune imprinting in the influenza ferret model. Vaccines.

[110] Davis A.S., Taubenberger J.K., Bray M. (2015). The use of nonhuman primates in research on seasonal, pandemic and avian influenza, 1893–2014. Antivir. Res..

[111] Moncla L.H., Ross T.M., Dinis J.M., Weinfurter J.T., Mortimer T.D., Schultz-Darken N., Brunner K., Iii S.V.C., Boettcher C., Post J. (2013). A novel nonhuman primate model for influenza transmission. PLoS ONE.

[112] Rimmelzwaan G.F., Kuiken T., van Amerongen G., Bestebroer T.M., Fouchier R.A.M., Osterhaus A.D.M.E. (2001). Pathogenesis of influenza A (H5N1) virus infection in a primate model. J. Virol..

[113] Baskin C.R., Bielefeldt-Ohmann H., Tumpey T.M., Sabourin P.J., Long J.P., García-Sastre A., Tolnay A.-E., Albrecht R., Pyles J.A., Olson P.H. (2009). Early and sustained innate immune response defines pathology and death in nonhuman primates infected by highly pathogenic influenza virus. Proc. Natl. Acad. Sci. USA.

[114] Rimmelzwaan G.F., Baars M., van Beek R., van Amerongen G., Lövgren-Bengtsson K., Claas E.C., Osterhaus A.D. (1997). Induction of protective immunity against influenza virus in a macaque model: Comparison of conventional and iscom vaccines. J. Gen. Virol..

[115] Batchelder C.A., Duru N., Lee C.I., Baker C.A.R., Swainson L., Mccune J.M., Tarantal A.F. (2014). Myeloid-lymphoid ontogeny in the rhesus monkey (Macaca mulatta). Anat. Rec. (Hoboken).

[116] Merino K.M., Slisarenko N., Taylor J.M., Falkenstein K.P., Gilbert M.H., Bohm R.P., Blanchard J.L., Ardeshir A., Didier E.S., Kim W.-K. (2020). Clinical and immunological metrics during pediatric rhesus macaque development. Front. Pediatr..

[117] Oxford K.L., Dela Pena-Ponce M.G.A., Jensen K., Eberhardt M.K., Spinner A., Van Rompay K.K., Rigdon J., Mollan K.R., Krishnan V.V., Hudgens M.G. (2015). The interplay between immune maturation, age, chronic viral infection and environment. Immun. Ageing.

[118] van Riel D., Munster V.J., de Wit E., Rimmelzwaan G.F., Fouchier R.A.M., Osterhaus A.D.M.E., Kuiken T. (2007). Human and avian influenza viruses target different cells in the lower respiratory tract of humans and other mammals. Am. J. Pathol..

[119] Weinfurter J.T., Brunner K., Iii S.V.C., Li C., Broman K.W., Kawaoka Y., Friedrich T.C. (2011). Cross-reactive T cells are involved in rapid clearance of 2009 pandemic H1N1 influenza virus in nonhuman primates. PLoS Pathog..

[120] Cillóniz C., Shinya K., Peng X., Korth M.J., Proll S.C., Aicher L.D., Carter V.S., Chang J.H., Kobasa D., Feldmann F. (2009). Lethal Influenza virus infection in macaques is associated with early dysregulation of inflammatory related genes. PLoS Pathog..

[121] Kobasa D., Jones S.M., Shinya K., Kash J.C., Copps J., Ebihara H., Hatta Y., Hyun Kim J., Halfmann P., Hatta M. (2007). Aberrant innate immune response in lethal infection of macaques with the 1918 influenza virus. Nature.

[122] Carroll T.D., Matzinger S.R., Genescà M., Fritts L., Colòn R., McChesney M.B., Miller C.J. (2008). Interferon-induced expression of MxA in the Respiratory tract of rhesus macaques is suppressed by influenza virus replication. J. Immunol..

[123] Kim J.R., Holbrook B.C., Hayward S.L., Blevins L.K., Jorgensen M.J., Kock N.D., Paris K.D., D’Agostino R.B., Aycock S.T., Mizel S.B. (2015). Inclusion of flagellin during vaccination against influenza enhances recall responses in nonhuman primate neonates. J. Virol..

[124] Holbrook B.C., Hayward S.L., Blevins L.K., Kock N., Aycock T., Parks G.D., Alexander-Miller M.A. (2015). Nonhuman primate infants have an impaired respiratory but not systemic IgG antibody response following influenza virus infection. Virology.

[125] Holbrook B.C., D’Agostino R.B., Parks G.D., Alexander-Miller M.A. (2016). Adjuvanting an inactivated influenza vaccine with flagellin improves the function and quantity of the long-term antibody response in a nonhuman primate neonate model. Vaccine.

[126] Holbrook B.C., Aycock S.T., Machiele E., Clemens E., Gries D., Jorgensen M.J., Hadimani M.B., King S.B., Alexander-Miller M.A. (2018). An R848 adjuvanted influenza vaccine promotes early activation of B cells in the draining lymph nodes of non-human primate neonates. Immunology.

[127] Krammer F., Palese P. (2013). Influenza virus hemagglutinin stalk-based antibodies and vaccines. Curr. Opin. Virol..

[128] Bui H.-H., Peters B., Assarsson E., Mbawuike I., Sette A. (2007). Ab and T cell epitopes of influenza A virus, knowledge and opportunities. Proc. Natl. Acad. Sci. USA.

[129] Nachbagauer R., Wohlbold T.J., Hirsh A., Hai R., Sjursen H., Palese P., Cox R.J., Krammer F. (2014). Induction of broadly reactive anti-hemagglutinin stalk antibodies by an H5N1 vaccine in humans. J. Virol..

[130] Jazayeri S.D., Poh C.L. (2019). Development of universal influenza vaccines targeting conserved viral proteins. Vaccines.

[131] Kirchenbaum G.A., Carter D.M., Ross T.M. (2016). Sequential infection in ferrets with antigenically distinct seasonal H1N1 influenza viruses boosts hemagglutinin stalk-specific antibodies. J. Virol..

